# An Unusual Gastrointestinal Presentation of Sjogren's Syndrome: A Case Report

**DOI:** 10.1002/ccr3.71112

**Published:** 2025-10-22

**Authors:** Oudai Sahwan, Fares Jamal, Amani Elshaer, Lin Batha, Tala Shahin, Michael M. Pham

**Affiliations:** ^1^ Division of Hematology and Oncology Mayo Clinic Phoenix Arizona USA; ^2^ Division of Internal Medicine Mayo Clinic Phoenix Arizona USA; ^3^ College of Medicine Alfaisal University Riyadh Kingdom of Saudi Arabia; ^4^ Division of Rheumatology Mayo Clinic Phoenix Arizona USA

**Keywords:** autoimmune disorder, gastrointestinal involvement, immunosuppressive therapy, Sjögren's syndrome

## Abstract

Sjögren's syndrome (SS) is a chronic autoimmune disorder characterized by lymphocytic infiltration and destruction of exocrine glands, commonly involving the salivary and lacrimal glands, leading to dry mouth and eyes. While SS primarily affects the upper gastrointestinal tract, this case presents a rare small intestine involvement. This patient was diagnosed with SS in 2018 with positive antinuclear antibodies (ANA), rheumatoid factor (RF), SSA antibodies, and salivary lip gland biopsy. By 2021, she developed unusual cyclic episodes of nausea, vomiting, and poor oral intake, resulting in significant weight loss and recurrent hospitalizations. She had frequent hospitalizations due to her recurrent symptoms. Evaluations included esophagogastroduodenoscopy (EGD) and colonoscopy, but pathology was nondiagnostic. Several abdominal and pelvic computed tomography (CT) scans demonstrated bladder edema, pancolitis, and terminal ileitis with mucosal hyperenhancement of the bowel and moderate ascites, likely inflammatory. Her gastric emptying study showed hypermotility. Multiple teams, including Rheumatology, Allergy/Immunology, Gastroenterology, Hematology, and Surgery, were involved. C1 esterase antigen was normal, ruling out hereditary angioedema. PET‐CT scan did not show lymphadenopathy or significant inflammation. Full biopsy for definitive diagnosis was deferred due to concerns of decreased healing with an edematous intestine. She was trialed on Icatibant without benefit. Differentials included lupus peritonitis, protein‐losing enteropathy, and SS intestinal lymphoma. She was discharged on a trial of empiric high‐dose steroids with Rheumatology follow‐up. Symptoms recurred with steroid tapering. Her complement levels decreased with flares and increased with treatment. Rituximab was added, leading to steady clinical improvement and reduced hospitalizations. Bowel involvement has been predominantly described in lupus, often referred to as lupus enteritis or bowel vasculitis. To our knowledge, this represents the first reported case of Sjögren's syndrome. Bowel biopsies are not typically subjected to routine immunohistochemical staining. Consequently, standard testing may be limited in detecting autoimmune processes. This case highlights the diagnostic challenges of SS‐related gastrointestinal manifestations and underscores the importance of considering autoimmune enteropathy, particularly in patients who respond to immunosuppressive therapy.

**Abbreviations**
ANA: antinuclear antibodies
CT: computed tomography
EGD: esophagogastroduodenoscopy
ITP: immune thrombocytopenic purpura
PET: positron emission tomography
PMNs: polymorphonuclear cells
RF: rheumatoid factor
SS: Sjögren's syndrome

**Summary**

- Sjögren's syndrome can present with recurrent, unexplained gastrointestinal symptoms mimicking other autoimmune diseases; clinicians should consider Sjögren's syndrome when standard GI workups are inconclusive.

## 1 Introduction

Sjögren's syndrome (SS) is a chronic multisystem autoimmune illness of the exocrine glands that is distinguished by lymphocytic infiltration of the afflicted gland [1]. It primarily affects females in a 9:1 female‐to‐male ratio, occurring across all ages but most commonly in the fourth and sixth decades [2]. The pathogenesis involves lymphocytic infiltration and destruction of the glandular tissue, thus reducing the glandular secretion. Salivary and lacrimal glands are the most frequently involved, with significant loss of their secretory function resulting in oral and eye dryness [2, 3]. Systemic manifestations, occurring in approximately 30% to 40% of patients, can affect the lungs, kidneys, vasculature, and blood [2, 4]. The gastrointestinal manifestation of SS primarily affects the upper gastrointestinal tract, resulting in dyspepsia, chronic atrophic gastritis, and primary biliary cirrhosis. Our case describes an unusual SS case with small intestine involvement, which is considered to be very rare [5].

## 2 Case History and Examination

**FIGURE 1:**
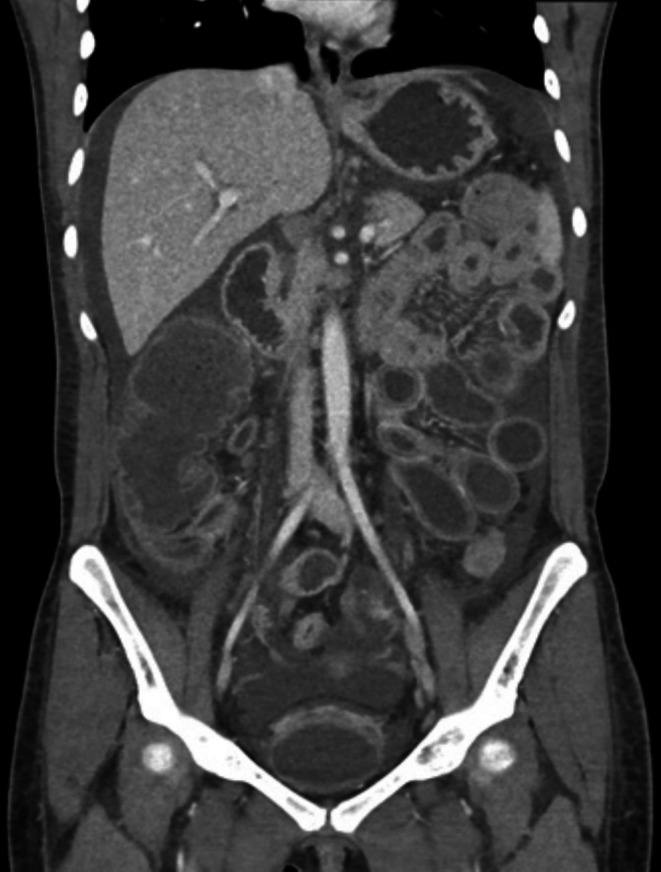
CT scan prior to therapy: bladder edema, pancolitis, and terminal ileitis with mucosal hyperenhancement of the bowel and moderate ascites.

This 27‐year‐old woman was diagnosed with SS based on elevated antinuclear antibodies (ANA), rheumatoid factor (RF), positive anti‐SSA antibodies, and lip biopsy. Lupus‐specific serologies, including anti‐dsDNA and anti‐Smith antibodies, were negative, making systemic lupus erythematosus (SLE) unlikely. She initially received hydroxychloroquine but discontinued it after 1–2 months due to severe headaches. Two years after her diagnosis, she began experiencing cyclic episodes of nausea and vomiting, resulting in an inability to tolerate oral intake and significant weight loss, which prompted her visit to our emergency department. A computed tomography (CT) scan of the abdomen and pelvis raised concerns about pancolitis and terminal ileitis (Figure 1). Due to concern for an inflammatory etiology, the gastroenterology team was consulted, and the patient underwent esophagogastroduodenoscopy (EGD) and colonoscopy. EGD showed grade A esophagitis, erythematous mucosa, gastric mucosal atrophy, and a normal duodenum. Colonoscopy showed small nonbleeding internal hemorrhoids; otherwise, it was unremarkable. Her biopsies were unrevealing. She was subsequently discharged on pantoprazole, bowel regimen, and anti‐nausea medications.

## 3 Differential Diagnosis, Investigations, and Treatment

A month later, she was readmitted with similar complaints. A repeat CT scan revealed the progression of colitis with increased bowel edema. She developed peritoneal signs and underwent paracentesis, which showed an elevated total nucleated cell count with increased polymorphonuclear cells (PMNs). She was treated with a 5‐day course of intravenous antibiotics. The workup during this admission included a transthoracic echocardiogram, liver biopsy, and gastrointestinal pathogen panel, all of which were unremarkable. The CT angiography of the abdomen showed widely patent celiac, superior mesenteric, and inferior mesenteric arteries, as well as widely patent mesenteric, splenic, and portal veins. Her stool test was positive for *Helicobacter pylori* . She was discharged with a treatment plan for *Helicobacter pylori* , including metronidazole, bismuth, doxycycline, and a proton pump inhibitor.

**FIGURE 2:**
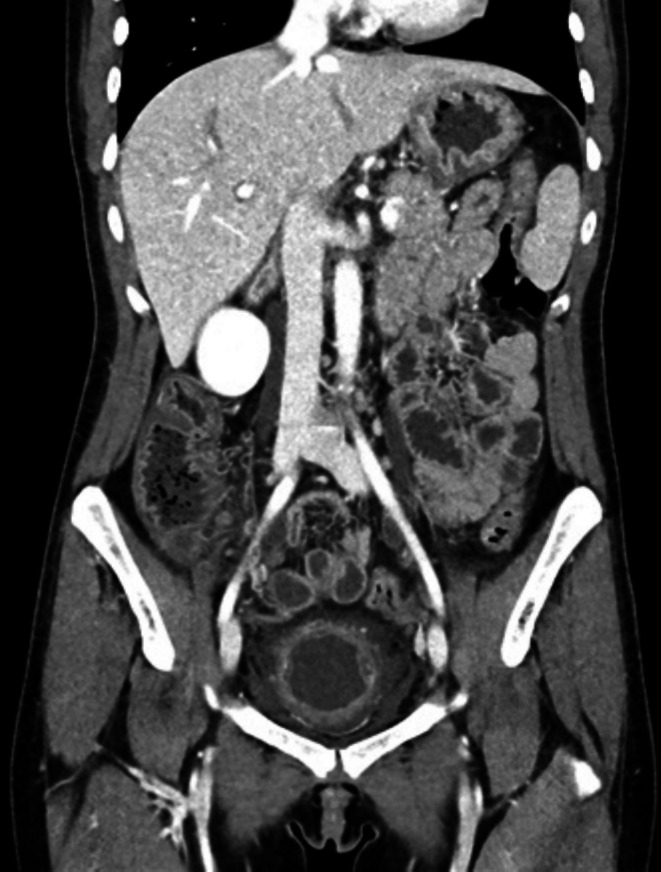
CT scan after therapy: decreased mural thickening, decreased ascites, and decreased bladder wall edema.

Unfortunately, a month after discharge, her symptoms recurred, leading to another hospitalization. A CT scan during this admission revealed marked intestinal dilation with improving ascites. A differential diagnosis of intestinal angioedema was proposed by the Allergy and Immunology department. Due to concern for angioedema and reduced complement levels, her C1 esterase was checked, which was within normal limits. A full‐thickness biopsy with partial bowel resection was considered, but general surgery was deemed too risky due to significant edema. She underwent a positron emission tomography (PET) scan, which was unrevealing. She was started on an empirical trial of Icatibant as a diagnostic test to explore whether the bradykinin pathway was involved. This made no meaningful difference in her symptoms or CT imaging. After discussion with multiple services, it was decided to initiate a trial of high‐dose steroids, which led to a gradual improvement in symptoms and imaging (Figure 2). She was subsequently discharged on a tapering regimen of prednisone, 40 mg daily.

She had just tapered her prednisone dose from 10 mg to 5 mg when she began experiencing a recurrence of her cyclic pain, nausea, and emesis, prompting a visit to the emergency department. A CT scan revealed nonspecific gastritis and colitis with gastric wall and distal colonic edema, showing improvement from previous imaging. The Hepatology team considered porphyria as a possible diagnosis, and a genetic panel revealed abnormalities, with further workup recommended. Laboratory workup revealed decreased complement levels and thrombocytopenia, both of which improved with steroid treatment. Hematology and Oncology noted that a repeat evaluation for porphyria was nondiagnostic and instead attributed her thrombocytopenia to immune thrombocytopenic purpura (ITP). Given that her symptoms appeared to correlate with steroid tapering, it was recommended that she be discharged on prednisone 60 mg daily. Additionally, Rheumatology recommended initiating rituximab to treat her unspecified autoimmune condition.

## 4 Conclusion and Results (Outcome and Follow‐Up)

After starting rituximab, the patient gradually improved and had no further hospitalizations. Her symptoms became more stable, although she continued to experience some abdominal pain and nausea, especially postprandially, along with mild fatigue. With rituximab, she was able to taper her steroid dosage slowly without major setbacks. As of now, her symptoms have significantly improved, though not completely resolved, and she remains stable on a reduced daily prednisone dose of 5 mg.

## 5 Discussion

This case may represent the first reported instance of bowel enteritis in SS, a condition more commonly associated with lupus. While enteritis is a well‐documented manifestation in lupus, its occurrence in SS is uncommon, making this a unique and noteworthy finding. Although lupus enteritis was considered in the differential, our patient had negative anti‐dsDNA and anti‐Smith antibodies and lacked systemic features of lupus such as nephritis, cutaneous involvement, or serositis. These findings, together with her established SS diagnosis and steroid/rituximab responsiveness, make lupus enteritis unlikely. This case highlights the need for further investigation into potential gastrointestinal manifestations of SS and broadens our understanding of its systemic involvement. The patient's recurrent hospitalizations and extensive diagnostic workup illustrate the diagnostic complexity of such atypical presentations. While her biopsy results were inconclusive, imaging studies revealed severe inflammation characterized by bladder edema, pancolitis, terminal ileitis, and significant ascites. Her marked improvement with high‐dose steroids and rituximab further illustrates the importance of immunosuppressive therapies in managing gastrointestinal complications of SS, reinforcing the need for aggressive treatment in severe cases.

This case is consistent with previously reported instances of rare gastrointestinal involvement in Crooks and Zweiman, who described a patient with severe nausea, vomiting, and vague abdominal pain, later attributed to renal tubular acidosis, another rare SS manifestation. The patient's symptoms responded favorably to steroid therapy [6]. Similarly, Xu et al. documented a case of a 66‐year‐old man whose primary complaint was chronic refractory diarrhea [7]. Despite normal endoscopic and imaging findings, his diagnosis was confirmed by positive anti‐SSA antibodies, Schirmer's test, and a salivary gland biopsy [7]. Glucocorticoid treatment led to substantial improvement, demonstrating the critical role of immunosuppression in managing gastrointestinal manifestations of SS.

Another multicenter randomized cohort study by Melchor et al. provides critical insights into the prevalence of digestive disorders in patients diagnosed with Sjögren's syndrome (SS) [8]. Among the 437 patients studied, nearly a third (29.5%) experienced atrophic gastritis, 16.9% had esophageal motility dysfunction, 4.2% developed lymphocytic colitis, and 21.1% were diagnosed with autoimmune hepatitis. Furthermore, the study identified a significant correlation between gastrointestinal symptoms and female patients (*p* = 0.0009), particularly those with comorbid autoimmune disorders such as hypothyroidism and C3 hypocomplementemia (*p* = 0.040) [8]. These findings show the diverse and systemic nature of SS, highlighting its potential to cause significant gastrointestinal complications.

Sobhani et al. also reported significant remission in a patient presenting with chronic diarrhea, malabsorption, and weight loss following a diagnosis of SS [9]. The patient had diffuse T lymphocytic infiltration throughout the gastrointestinal tract, with symptoms dramatically improving after immunosuppressive therapy. This case, along with others, highlights the importance of early recognition and appropriate treatment of gastrointestinal involvement in SS.

Effective management of these patients relies on prompt diagnosis and the use of immunosuppressive therapies, such as rituximab and high‐dose steroids, to prevent recurrent hospitalizations and manage severe manifestations. This emphasizes the broader importance of considering SS in differential diagnoses when gastrointestinal symptoms are present and the potential benefits of immunosuppressive treatment in achieving long‐term symptom control.

## Author Contributions

**Oudai Sahwan:** conceptualization, writing – original draft, writing – review and editing. **Fares Jamal:** writing – original draft, writing – review and editing. **Amani Elshaer:** writing – review and editing. **Lin Batha:** writing – original draft, writing – review and editing. **Tala Shahin:** writing – review and editing. **Michael M. Pham:** supervision, writing – review and editing.

## Ethics Statement

The authors have nothing to report.

## Consent

Written consent was obtained from the patient for publication.

## Conflicts of Interest

The authors declare no conflicts of interest.

## Data Availability

The authors have nothing to report.
